# Surveillance of avian influenza viruses from 2014 to 2018 in South Korea

**DOI:** 10.1038/s41598-023-35365-4

**Published:** 2023-05-24

**Authors:** Erica Españo, Sang-Mu Shim, Eun-Jung Song, Jeong-Hyun Nam, Seo-Hee Jeong, Bill Thaddeus Padasas, Sang-Hyun Kim, Jeong-Ki Kim

**Affiliations:** 1 Department of Pharmacy, Korea University College of Pharmacy, Sejong, 30019 Republic of Korea; 2 Division of Acute Viral Diseases, Center for Emerging Virus Research, National Institute of Infectious Diseases, National Institute of Health, Cheongju, Chungbuk 28159 Republic of Korea; 3 Laboratory Animal Medicine, College of Veterinary Medicine, Chonnam National University, Gwangju, 61186 Republic of Korea; 4 Division of Vaccine Clinical Research, Center for Vaccine Research, National Institute of Infectious Diseases, National Institute of Health, Cheongju, Chungbuk 28159 Republic of Korea

**Keywords:** Influenza virus, Virology, Phylogenetics

## Abstract

Surveillance of influenza A viruses (IAVs) among migratory waterfowl is a first step in understanding the ecology, biology, and pathogenicity of IAVs. As part of the nationwide surveillance effort for IAVs in fowl in South Korea, we collected environmental fecal samples in different migratory bird stopover sites in South Korea during the winter seasons within November 2014 through January 2018. We collected a total of 6758 fecal samples, 75 of which were positive for IAV (1.11% positivity). Prevalence of IAVs varied per site and per year. Based on sequencing, the most prevalent hemagglutinin (HA) subtypes were H1, H6, and H5, and the most prevalent neuraminidase (NA) subtypes were N1, N3, and N2. Phylogenetic analyses showed that the genes we isolated clustered with reported isolates collected from other locations along the East Asian-Australasian Flyway. All the H5 and H7 isolates collected in this study were of low pathogenicity. None of the N1 and N2 genes carried amino acid markers of resistance against NA inhibitors. The winter 2016–2017 subset were primarily borne by migratory geese (*Anser* spp.). These results suggest that majority of the IAVs circulating among migratory wild fowl in South Korea in 2014–2018 were of low pathogenicity.

## Introduction

Wild waterfowl (Anseriformes) and shorebirds (Charadriiformes) are the known reservoir of influenza A viruses (IAVs) of the *Orthomyxoviridae* family^1^. Barring the two hemagglutinin (HA; H17 and H18) and two neuraminidase (NA; N10 and N11) subtypes recently discovered in bats, these birds carry all known IAV subtypes (H1–H16 and N1–N9). Wild waterfowl and shorebirds can carry IAV genotypes across flyways, and, to a lesser degree, across oceans^2^. Open farming allows the sharing of food and water resources between wild and domestic waterfowl that may lead to the transmission of IAVs from wild to domestic birds^3^. Domestic waterfowl, especially ducks, can then act as intermediate hosts for the transmission of IAVs to poultry, primarily chicken (Galliformes).

Certain H5 and H7 IAV strains can cause severe disease in chicken and domestic ducks and are thus called highly pathogenic avian influenza viruses (HPAIVs)^4^. Poultry infected with HPAIVs exhibit severe clinical signs, including paralysis and sudden death, with up to 100% mortality during outbreaks. Because HPAIVs can rapidly spread within a farm and to adjacent farms, HPAIV outbreak control requires culling all birds in affected farms, thereby causing huge economic losses^5^. The effects of HPAIVs to wild birds are traditionally considered mild. However, reports of HPAIV-related deaths in wild birds have been growing in number, warranting the reassessment of the effects of HPAIVs to wild birds, especially to protected species^6,7^. HPAIV outbreaks among poultry also expose poultry farm workers to the virus and can cause a range of mild to severe flu symptoms in humans, with approximately 50% case fatality for the H5N1 HPAIV subtype^8^. Although HPAIVs have not yet caused sustained chains of human-to-human transmission, they are regarded as potential pandemic threats. Meanwhile, low pathogenic avian influenza viruses (LPAIVs) are also known to contribute genetic material to pandemic influenza virus strains and are thus considered precursors of future pandemic influenza viruses^9^. Genetic material from LPAIVs can also be used to design vaccines for poultry. Therefore, the surveillance of circulating avian influenza viruses, including LPAIVs, is imperative to the understanding of IAV ecology, which impacts both animal and human health.

South Korea has experienced HPAIV outbreaks that have been linked to circulating HPAIVs or precursors carried by migratory wild birds^10–12^. In 2008, the government of South Korea implemented a national influenza virus surveillance program to identify IAVs in birds that have the potential to cause HPAIV outbreaks among poultry and may be a threat to humans^13^. Here, we report the isolation and phylogenetic analyses of avian influenza viruses from fecal matter of wild birds collected in migratory bird stopover sites in South Korea in winters throughout November 2014 to January 2018 as part of the nationwide surveillance program.

## Results

### Prevalence of avian influenza viruses in South Korea in the winters of 2014 to 2018

**Figure 1:**
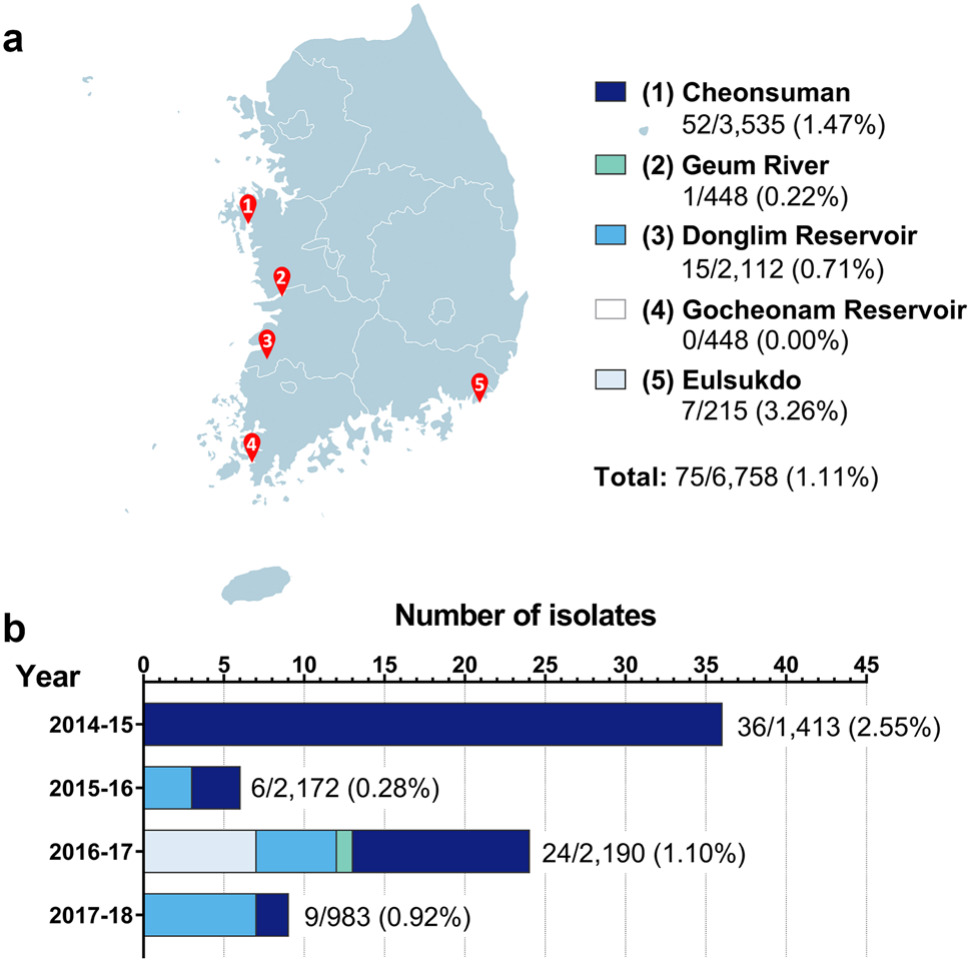
Isolation of influenza A viruses (IAVs) from fecal matter collected at five migratory bird stopover sites in South Korea (winters of November 2014 to January 2018). **(a)** Fecal samples were collected at five migratory bird stopover sites during the winter seasons from November 2014 to January 2018. The number of samples that tested positive for IAV over the total number of samples collected per site are indicated. Numbers in parentheses indicate percentage of IAV-positive samples per site. **(b)** Number of samples that tested positive for IAV per year over the total number of samples collected per year are indicated. Numbers in parentheses indicate percentage of IAV-positive samples per year. Distribution of positive samples per site per year are also shown (color-matched with **a**).

Samples of fresh bird fecal matter were collected from different sites in South Korea (Fig. 1a) during winters within November 2014 to January 2018. Each winter was defined as November to January of two consecutive years (i.e., November 2014 to January 2015 was defined as the winter of 2014–2015). The sampling sites are known stopover points of migratory birds, especially those coming from Far East Siberia during the annual winter migration along the East Asian-Australasian Flyway (EAAF). As in our previous study that covered winters of 2009 to 2013^14^, the stopover sites were selected for their proximity to previous sites of HPAI outbreaks. A total of 6758 fecal samples were collected throughout the study (Fig. 1a, Table 1). Of these, 75 samples tested positive for IAV through the standard chicken allantoic fluid isolation method and RT-PCR, indicating isolation from 1.11% of the total number of samples for the entire duration of the study. Percent IAV-positivity of samples were highly variable per site and per year (Fig. 1a,b, Table 1), with the winter of 2014–2015 having the highest percent positivity (2.55%), although all the IAV-positive samples that season came from only one of two sampling sites. The winter season with the lowest percent IAV-positivity was 2015–2016 (0.28%), despite a relatively large number of collected samples. Not all sites yielded positive samples, as no IAV was isolated from wild bird fecal matter collected at Gocheonam Reservoir in Jeonnam Province, in contrast with our 2009–2013 study where we reported isolation of IAVs from this site.

**Table 1:** Number of isolates of avian influenza viruses per year and per site in South Korea in winters within November 2014 to January 2018.

Site	2014 to 2015		2015 to 2016		2016 to 2017		2017 to 2018		Total		% Positive per site over total positives (75)
	Samples	Positives (%)*	Samples	Positives (%)*	Samples	Positives (%)*	Samples	Positives (%)*	Samples	Positives per site (%)	
Cheonsuman	1330	36 (2.71%)	1500	3 (0.20%)	445	11 (2.47%)	260	2 (0.77%)	3535	52 (1.47%)	69.33
Eulsukdo	83	0 (0)	–	–	132	7 (5.30%)	–	–	215	7 (3.26%)	9.33
Geum River	–	–	336	0 (0)	112	1 (0.89%)	–	–	448	1 (0.22%)	1.33
Donglim Reservoir	–	–	336	3 (0.89%)	1053	5 (0.47%)	723	7 (0.97%)	2112	15 (0.71%)	20.00
Geocheonam Reservoir	–	–	–	–	448	0 (0)	–	–	448	0 (0)	0.00
Total	1413	36 (2.55%)	2172	6 (0.28%)	2190	24 (1.1%)	983	9 (0.92%)	6758	75 (1.11%)	100.00

*Percent positivity per year are calculated based on the total number of isolates that year.

### HA and NA subtype prevalence and combinations

**Figure 2:**
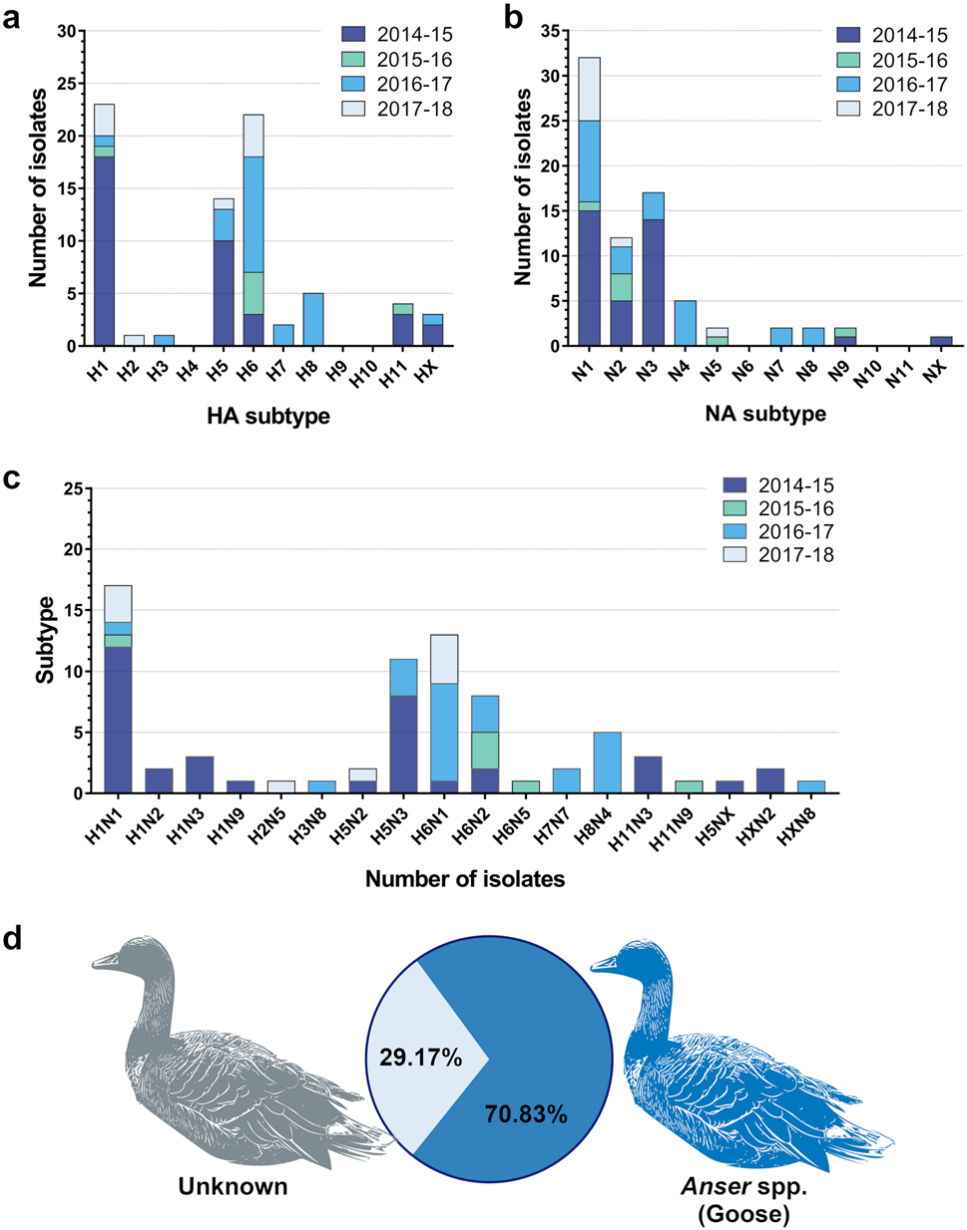
Subtypes of influenza A isolates collected in migratory bird stopover sites in South Korea during winter from November 2014 to January 2018. **(a)** Hemagglutinin (HA), **(b)** neuraminidase (NA) subtypes, and **(c)** combinations thereof of all influenza A virus isolates collected over the entire surveillance period. **(d)** Host species carrying the influenza A virus isolates for winter 2016–2017 were determined through sequencing of the *COX1* gene amplified from DNA isolated from the fecal samples.

Not all HA and NA subtypes and combinations thereof were represented over the duration of the study (Fig. 2a–c, Supplementary Table S1–S3). For the study period, the most common HA subtypes were H1 (n = 23; 30.67%), H6 (n = 22; 29.33%), and H5 (n = 14; 18.67%) (Fig. 2a, Supplementary Table S1). No virus of the H4, H9, or H10 subtype was identified. However, we were unable to identify the HA subtypes of 3 samples (HX). Different years also had different dominant HA subtypes; the H1 subtype was the most prevalent in 2014–2015, while the H6 subtype was the most prevalent in the succeeding years. The H5 subtype was also among the most prevalent subtypes in most years, except in 2015–2016, when no H5-subtype IAV was isolated.

For the duration of the study, the most common NA subtypes were N1 (n = 32; 42.67%), N3 (n = 17; 22.67%), and N2 (n = 12; 16.00%) (Fig. 2b, Supplementary Table S2). No virus belonging to the N6, N10, and N11 subtype was isolated; however, the NA subtype of one sample could not be identified (NX). Isolates belonging to the N1 subtype were the most prevalent in the winters of 2014–2015, 2016–2017, and 2017–2018, whereas isolates of the N2 subtype were the most prevalent during the winter of 2015–2016. The most common subtype combinations were H1N1 (n = 17; 22.67%), H6N1 (n = 13; 17.33%), H5N3 (n = 11; 14.67), and H6N2 (n = 8; 10.67%) (Fig. 2c, Supplementary Table S3). The prevalent subtype combinations varied per year, with H1N1 as the most prevalent subtype in 2014–2015; H6N2 in 2015–2016; and H6N1 in 2016–2017 and 2017–2018.

### HA and NA sequence identities

The HA and NA sequences for the isolates were assembled to generate full and partial coding sequences. These sequences were deposited to GenBank (Supplementary Table S4) and subjected to BLASTn searches in both NCBI and GISAID to identify reported HA and NA isolates that were most similar to our isolates (Supplementary Table S5). As expected, our isolates matched with samples collected from either South Korea or nearby locations (China, Japan, and Mongolia), all of which are part of the EAAF. The closest sequence matches were isolated within similar periods of time (2014–2021). None of the H5 and H7 sequences matched with reported HPAIVs, suggesting that all isolated viruses in this study were LPAIVs. A closer look at the H5 sequences showed the lack of the multibasic HA cleavage site characteristic of HPAIVs (Supplementary Fig. S1a), further indicating that our H5 isolates were LPAI. However, all the H5 isolates carried reported markers for potential mammalian transmission (Supplementary Table S6) as indicated by the “Identify Sequence Features in Segments” tool in the Influenza Research Database. Specifically, majority of the H5 isolates carried the N171, A172, and P251 variations, which were all reported to enhance HA binding to the human ⍺2,6-linked sialic acid^15,16^. The algorithm did not report notable variations at the 226 amino acid position in the H5 isolates. Meanwhile, the HA amino acid sequences of the two H7 isolates in this study as well as the closely related A/crane/Kor/17 (H7N7) also lacked the multibasic cleavage site, further verifying that they were LPAIVs (Supplementary Fig. S1b).

### Host species identification for IAVs isolated in the winter of 2016–2017

To obtain a snapshot of the host that carried some of our isolates, we extracted genomic DNA from the IAV-positive fecal samples collected during the winter of 2016–2017 (n = 24). The cytochrome C oxidase 1 (*COI*) gene was amplified from genomic DNA extracted from the fecal matter of the winter 2016–2017 samples, and the sequences of these amplicons were used for identifying the host species using the Barcode of Life Data (BOLD) system. All IAV isolates were carried by members of the genus *Anser* (wild geese) of the family Anatidae in the order Anseriformes (Fig. 2d, Supplementary Table S7). We were unable to identify the host at the species level for all the samples owing to nearly identical *COI* sequences among members of the genus *Anser*. However, the top hits included *A. fabalis* (bean goose), *A. albifrons* (greater white-fronted goose), *A. anser* (greylag goose)*,* and *A. cygnoides* (swan goose)*.* Both *A. fabalis* and *A. albifrons* are among the most common annual visitors to South Korea during winter and are the most probable avian hosts for the isolated IAVs^15^. We were unable to sequence the *COI* genes of 7 out of 24 samples owing to the poor quality of DNA extracted from the fecal samples.

### Phylogenetic analyses of predominant HA subtypes

**Figure 3:**
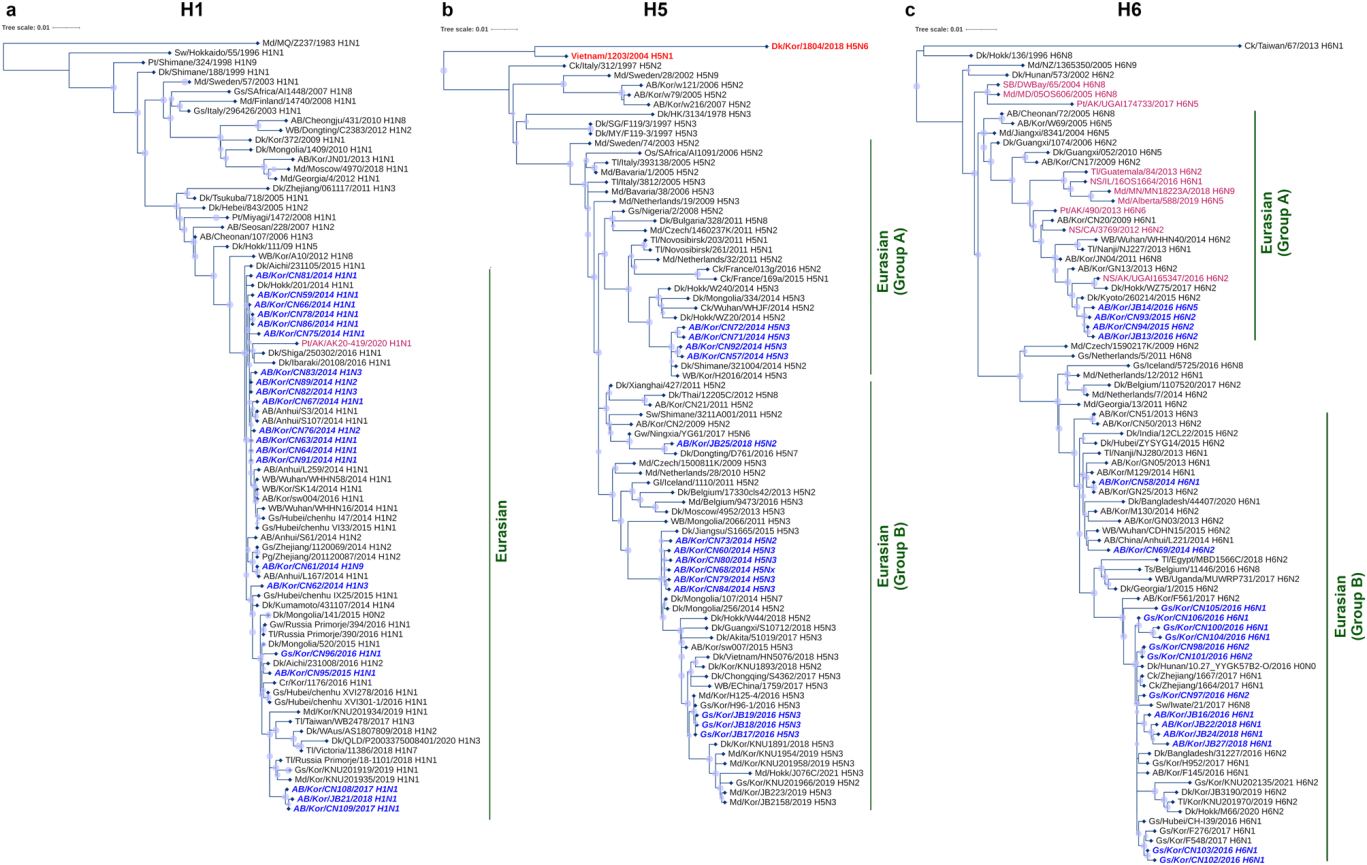
Phylogenetic analyses of the three most prevalent hemagglutinin subtypes collected in South Korea during winter from November 2014 to January 2018. Assembled full and partial** (a)** H1, **(b)** H5, and **(c)** H6 sequences of isolates (blue, bold-italic) compared with full coding sequences available at GenBank and GISAID. Isolates in bold red (**b**, H5 tree) indicate highly pathogenic avian influenza viruses (HPAI). Isolates collected from the Americas are labeled in magenta. Circles at nodes indicate transfer support values from 60 to 100%; the size of the circle is proportional to the bootstrap score. *AB* aquatic bird, *Ck* chicken, *Dk* duck, *Gs* goose, *Md* mallard, *Kor* Korea.

Full and partial sequences of the three most prevalent HA subtypes (H1, H5, H6) were compared with sequences pulled from GenBank and GISAID for phylogenetic analyses (Fig. 3). Our H1 isolates clustered with H1 samples that have been isolated from various locations along the EAAF including South Korea since 2004 (Fig. 3a). This indicates that the current H1 gene pool in South Korea has long been circulating in South Korea and other regions along EAAF. However, a recently reported sequence (2020) from Alaska also clustered with our isolates, indicating that the current EAAF H1 gene pool may have been recently carried over to a different continent by migratory birds either through long-distance migration or through interactions with other migratory bird species from the Pacific flyway.

In contrast, the H5 and H6 gene pools appear to be more dynamic than that of H1. Our H5 isolates clustered with H5 viruses that have long been circulating since at least 2002 all over Eurasia and Australia, covering all the Eurasian, African, and Australian flyways (Fig. 3b). Meanwhile, our H6 isolates clustered into two major groups: some isolates clustered with H6 samples from North America, while some isolates clustered with H6 samples primarily from Eurasia (Fig. 3c). However, considering that the North American sequences that clustered with our samples were obtained much later (2012 onwards; Fig. 3c, Eurasian Group A) than the earlier Asian samples in the same cluster (2004–2009; Fig. 3c, Eurasian Group A), the North American gene pool in this group may have arisen from the Eurasian gene pool.

### Phylogenetic analyses of predominant NA subtypes

**Figure 4:**
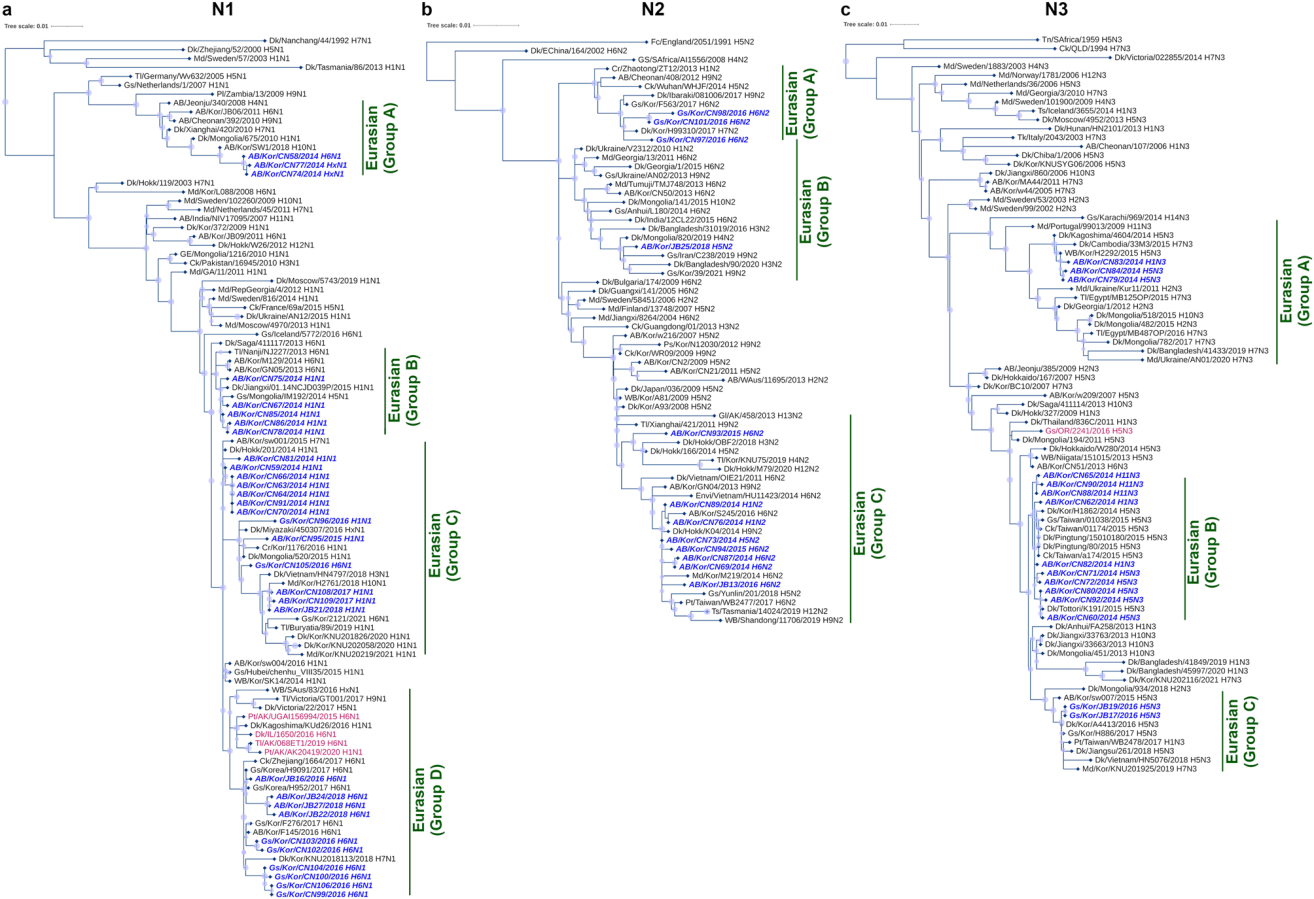
Phylogenetic analyses of the three most prevalent neuraminidase subtypes collected in South Korea during winter from November 2014 to January 2018. Assembled full and partial** (a)** N1, **(b)** N2, and **(c)** N3 sequences of isolates (blue, bold-italic) compared with full coding sequences available at GenBank and GISAID. Isolates collected from the Americas are labeled in magenta. Circles at nodes indicate transfer support values from 60 to 100%; the size of the circle is proportional to the bootstrap score. *AB* aquatic bird, *Ck* chicken, *Dk* duck, *Gs* goose, *Md* mallard, *Kor* Korea.

Full and partial sequences of the three most prevalent NA subtypes (N1, N2, and N3) were compared with sequences pulled from GenBank and GISAID for phylogenetic analyses (Fig. 4). The NA genes of our isolates clustered with isolates from the EAAF, and all belong to the Eurasian lineage. Notably, some of our N1 isolates clustered with samples isolated from North America, suggesting that some of the North American N1 gene in circulation have arisen from the Eurasian gene pool. N2 and N3 sequences of viruses isolated in North America also grouped with samples from Eurasia, indicating that Eurasian genes are carried by migratory birds to North America.

Because N1 and N2 subtype influenza viruses are the most associated with human illness and have the potential for reassortment with human influenza virus genes, we determined whether the N1 and N2 subtypes in this study were susceptible to approved NA inhibitors using the “Antiviral Resistance Risk Assessment” tool in the Influenza Research Database. All N1 isolates were found to be susceptible to oseltamivir, zanamivir, and peramivir. Meanwhile, all N2 isolates were found to be susceptible to oseltamivir and zanamivir.

## Discussion

Similar to our previous report on avian influenza surveillance from 2009 to 2013 in South Korea^14^, percent IAV-positivity of bird fecal samples and prevalent IAV subtypes varied per year and per site over the winters of November 2014 to January 2018. All H5 and H7 IAVs isolated during the surveillance period were LPAIVs and were highly similar to isolates obtained from different locations along the EAAF. As expected, most of the HA and NA genes of the isolates clustered with those from isolates obtained from other countries along the EAAF, including China, Japan, and Mongolia. Supporting recent reports that IAVs from wild birds do not always follow rigid flyway boundaries and that there is intercontinental movement of IAV gene pools carried by wild fowl, some of the HA and NA genes of our isolates also cluster with a number of North American isolates. In addition, as shown by the present study and previous results on the surveillance of avian influenza viruses in South Korea^14^, percent IAV-positivity of samples tends to fluctuate across the years; however, we currently have no specific explanation for this interannual variability.

Influenza host species identification using the *COI* gene amplified from genomic DNA extracted from bird droppings collected during the winter of 2016–2017 indicates that majority of the isolates during the season were carried by migratory geese (*Anser* spp.). Although we were unable to identify the host at the species level, we speculate that the isolated IAVs came from either the taiga bean goose (*A. fabalis*) or the greater white-fronted goose (*A. albifrons*), which are the most dominant goose species that winter in South Korea from breeding sites in Far East Siberia^17^. IAVs, including HPAIVs, have been previously isolated from these goose species in other studies in South Korea^13,18,19^; migratory geese are therefore presumed to contribute to HPAIV outbreaks in the country.

Similar to our surveillance study in previous years, IAVs of the H6 subtype were among the most frequently isolated during successive winters in South Korea. This is corroborated by studies that suggest that H6-subtype IAVs are the most frequently isolated subtypes from wild waterfowl especially in Asia^20^. A study from China reported that 11.07% of the isolates from wild birds in 2016–2017 were of the H6 subtype, making H6 the most predominant subtype in that study^21^. In our study, IAVs of the H6 subtype were likewise the most prevalent over the winter of 2016–2017, which may indicate similar distributions of IAV subtypes along the EAAF that year. Another study has reported that 49.44% (44/89) of isolates from wild birds in China along the EAAF over 2015–2019 were of the H6 subtype^22^. In our study, the H6-subtype IAVs were consistently isolated in all four years, suggesting its persistence in migratory waterfowl populations that traverse the EAAF. Recent studies from China suggest that H6NX viruses from wild birds, including A/*Anser fabalis*/China/Anhui/L221/2014 (H6N1), which is 99% identical to one of our isolates (A/Kor/CN69/2014 [H6N2]), can infect mice and replicate at high titers in mouse lungs without prior adaptation^23^. While H6-subtype IAVs are considered LPAI and are non-reportable, they may still cause damage to the poultry industry (~ 30% drop in egg production)^20^. Given the potential ability of H6-subtype IAVs to infect mammals, their persistence in the wild, and their effects on poultry, closer investigation of H6-subtype IAVs is warranted.

Meanwhile, in our study, viruses of the H1 subtype were predominant in the winter of 2014–2015, accounting for 50% (18/36) of the isolates that season. This is in line with a previous report of an H1NX outbreak among wild fowl in Anhui, China^24^ and from another study in bird wintering wetlands in Yangtze River, China^25^.

Despite H5 HPAI outbreaks in poultry in South Korea over the study period, we found no H5 HPAIV during our surveillance. However, H5-subtype LPAIVs, especially H5N3 LPAIVs, were among the most prevalent subtypes, particularly in the winters of 2014–2015 (27%) and 2016–2017 (12.5%). Another surveillance study in South Korea also reported the prevalence of H5 LPAIVs from wild bird habitats in 2015 (13.1%) and 2017 (14.4%), coinciding with our results^26^. In contrast to our findings, this previous study reported the isolation of H5-subtype LPAIVs in 2017 in the Jeonnam Province of South Korea, while we obtained no isolate from Gocheonam Reservoir in Jeonnam Province. However, the Jeonnam isolates of this study were obtained in December 2017, while we performed sampling in the same region in November 2016. The differences in isolation rates of H5 and other AIV isolates in this province may be due to sampling bias, as we only sampled a very small portion of a large migratory bird stopover site, and only performed sampling on a single day in a single winter season. Obtaining more avian fecal samples later in the season and on other years may have yielded different results.

All our H5 isolates carried markers of increased transmission to mammals. These markers include the N171and T172 variations which, when present together, have been reported to increase binding of the A/Viet Nam/1203/2004 (H5N1) HA to the human ⍺2,6-linked sialic acid without loss of binding to the avian ⍺2,3-linked sialic acid^15^. Another variation found in most of our isolates was P251, which has been reported to slightly increase the binding of the A/duck/Egypt/D1Br12/2007 (H5N1) HA binding to ⍺2,6-linked sialic acid^16^. The L226 variation was not present in any of our H5 isolates. These findings suggest that H5 genotypes circulating in wild birds passing through South Korea and adjacent countries continue to gain potential to infect mammals. The persistence of these H5 sequences in the wild increases their chances of reassortment with other IAV genes that may lead to eventual crossing to the mammalian species barrier and perhaps to sustained transmission among mammals including humans. Considering that H5 LPAIVs have been reported as precursors of HPAIVs, continued monitoring of H5 LPAIVs is needed to guide mitigation and control measures for H5 IAV infections in both wild and domestic fowl.

Only 2 fecal samples (2.6% of total) yielded H7-subtype isolates, both of which were H7N7 LPAIVs. Interestingly, both the HA and NA genes of the isolates were highly identical to those of an H7N7 isolate from a red-crowned crane (A/red-crowned crane/Korea/H1026/2017) in a zoo in South Korea isolated in March 2017^27^. Our H7N7 samples were collected in January 2017, placing this particular H7N7 gene pool in South Korea earlier than closely related H7N7 isolates collected from migratory mallards in February to March of 2017. Notably, H7 isolation was more frequent in the other study (43.6% of all isolates in early 2017) than in ours (8.3%), possibly owing to differences in sample timing, host species, and sampling method^28^. Regardless, transmission of H7 IAVs from migratory fowl to other bird species was highlighted by the isolation of the H7N7 isolate in the captive crane, which further emphasizes the likelihood of transmission of H7 IAVs from migratory wild birds to domestic birds.

As in previous years of our study, the IAV isolation rate was variable per year. This may be a result of changes in seasonal temperatures across different years. Migration of fowl, including bean geese and white-fronted geese, is highly affected by temperatures. The timing of bird migration to wintering sites may change based on temperatures in both the breeding and wintering sites^29^. This would then affect the composition of migratory fowl populations at stopover sites and, in turn, affect observed IAV prevalence. Environmental temperatures also affect the persistence of IAVs in aquatic environments and may affect the maintenance of IAVs in wild birds^30^; changes in temperatures across the years would then alter the prevalence of IAV infection in wild birds. Thus, the timing of IAV surveillance studies such as this should take into account potential variabilities in environmental temperatures and timing of bird migration to be able to obtain a more comprehensive view of IAV prevalence in breeding, stopover, or wintering sites.

One limitation of our study is that, at the time of writing, the sequences we report are already at least five years old. Several studies have already reported more recent data (2019 to 2022) on circulating avian influenza viruses among wildfowl that visit South Korea, although most have focused on H5-, H7-, and H9-subtype influenza viruses^19,31–33^. Given the continued threat of AIVs to both animal and human health, large amounts of genetic data are important to allow us to track the movement of avian influenza virus gene pools across time and geographic locations. Ensuring that sequences of influenza viruses over large periods of time are reported will fill in potential gaps of information in tracing back the evolution of future avian-origin influenza virus pandemics. This has been highlighted by the recent coronavirus disease (COVID-19) pandemic, where, owing to very few surveillance studies in the past and very little existing genetic information on severe acute respiratory syndrome (SARS)-related viruses prior to the pandemic, scientists have difficulty in fully understanding the emergence of SARS coronavirus 2 (SARS-CoV-2). Global public health will undoubtedly benefit from all available genetic information on AIVs especially within the past couple of decades, as these would be the first resources to investigate should an avian-origin pandemic arise. It would also allow us to further understand the dynamics of the continued spread and circulation of avian influenza viruses in the wild in the hope of preparing for large outbreaks in both wild and domestic birds.

Additionally, most AIV surveillance studies in South Korea focus on H5-, H7-, and H9-subtype influenza viruses. While these subtypes pose the highest and most immediate risk to veterinary and human health, other subtypes of AIVs may contribute genetic material to future threats, and, in rare cases, cause disease and death in humans^34^. Thus, we believe that long-term surveillance studies on IAVs that include LPAIVs and HPAIVs and the reportable and non-reportable subtypes can provide the scientific community a wealth of genetic information that can be utilized when the need arises.

All in all, in this study, we were able to collect a total of 6758 bird fecal samples from five migratory bird stopover sites in South Korea during the winters of 2014 to 2018 (November 2014 to January 2018). Of these, 75 (1.11%) tested positive for IAVs based on egg isolation and RT-PCR. The most common HA subtypes were H1, H6, and H5, and the most common NA subtypes were N1, N3, and N2. Based on sequence analysis, all isolates, including the H5- and H7-subtypes, were LPAIVs and were most similar to isolates collected along the EAAF. As in previous years, isolation rates and predominant subtypes varied per site and per year. Although we were unable to isolate HPAIVs from the bird fecal samples despite HPAIVs outbreaks in South Korea during the study period, this surveillance study provides insight into the dynamics of IAVs carried by migratory birds into South Korea.

## Methods

### Sample collection and virus isolation

Through the winter seasons of 2014 to 2018 (November 2014 to January 2018), a total of 6758 fecal samples were collected from stopover sites of wild migratory birds in South Korea. Fecal samples were stored in transport medium consisting of phosphate buffered saline (PBS) and glycerol (50%) with antibiotics (1000 U/ml penicillin G and polymyxin B, 500 U/ml nystatin, 250 µg/ml gentamicin, 60 µg/ml ofloxacin, and 200 µg/ml sulfamethoxazole) (Sigma-Aldrich, St. Louis, MO). The collected samples were stored at − 80 °C until analysis or egg passage.

The samples were suspended in antibiotic-supplemented PBS and inoculated into the allantoic cavity of 10-day-old embryonated chicken eggs^35^. The allantoic fluid was collected; turbid fluid was assumed to be contaminated with bacteria or fungi and was discarded. All viral isolates were collected from the first egg passage. Viral presence was identified through the hemagglutination assay using 0.5% chicken red blood cells.

### RT-PCR and sequence analysis

Viral gene amplification using samples that tested positive in the hemagglutination assay was performed as previously described^30^. Using the RNeasy Mini Kit (Qiagen, Valencia, CA), viral RNA was extracted from the hemagglutination-positive allantoic fluid. For HA and NA gene subtyping, one-step RT-PCR was carried out using the One Step RT-PCR Kit (Qiagen) with HA- or NA-specific universal primer sets designed and described in a previous study^36^. The RT-PCR reactions were set up according to manufacturer instructions. Each 50-μl reaction contained 1.5 μl of each primer (20 pmol/μl) and 2 μl of viral RNA (1 pg–2 µg). Reverse transcription was performed at 50 °C for 30 min, and standard PCR was performed with an initial denaturation at 94 °C for 10 min; followed by 35 cycles of 94 °C for 30 s, 56 °C for 30 s, and 72 °C for 2 min; and a final extension at 72 °C for 10 min. After purification with the QIAquick Gel Extraction Kit (Qiagen), the amplified gene segments were commercially sequenced at Cosmogenetech Co. (Seoul, South Korea). Sequencing was based on the Sanger sequencing technology using the high-throughput DNA Analyzer (Applied Biosystems 3730xl DNA Analyzer, Thermo-Fisher Scientific, Waltham, MA). Full-length coding sequences (HA: 1710 bp; NA: 1410 bp) were assembled using the Lasergene sequence analysis software package (DNASTAR, Madison, WI). A BLASTn query was performed in NCBI to identify the HA and NA subtypes of the isolates based on the obtained sequences and to identify isolates reported in GenBank that had the highest HA and NA coding sequence similarities to our isolates. The search results were supplemented with a BLAST analysis in GISAID especially for sequences that had hits with < 99% sequence identity on GenBank. Full and partial HA and NA sequences of the isolates were deposited to GenBank, and accession numbers are listed in Supplementary Table S1.

### Host identification using the *COI* gene

Genomic DNA was extracted from wild bird fecal droppings collected in winter 2016–2017 using the QIAamp DNA Stool Mini Kit (Qiagen) according to the manufacturer's protocol. The *COI* gene was isolated thru PCR amplification using bird-specific *COI* primers: forward primer: 5′-TTCTCCAACCACAAAGACATTGGCAC-3′, and reverse primer: 5′-ACGTGGGAGATAATTCCAAATCCTG-3′^37^, followed by purification using a QIAquick Gel Extraction kit (Qiagen). The amplified *COI* genes were commercially sequenced (Cosmogenetech), and the full sequences of the *COI* genes were assembled using the Lasergene analysis software. The host species were determined using the web version of BOLD SYSTEMS Identification Engine (https://www.boldsystems.org/index.php/IDS_OpenIdEngine), which is widely used for species-level identification^38^. The “All Barcode Records on BOLD” was selected as the search database, and the hits were determined based on % similarity with the assembled *COI* genes of the samples.

### Sequence alignment to determine sequence motifs

The H5 and H7 nucleotide sequences of the isolates were translated into amino acid sequences using the EMBOSS Transeq translation tool (https://www.ebi.ac.uk/Tools/st/emboss_transeq/). The amino acid sequences were aligned with sequences of reported HPAI H5 and H7 isolates using UGENE to determine whether the HA of the isolates had monobasic or multibasic cleavage sites.

### Phylogenetic analyses of HA and NA coding sequences

The assembled full and partial coding sequences of H1, H5, H6, N1, N2, and N3 isolates were aligned using CLUSTAL V^39^. Maximum likelihood phylogenetic trees were prepared through the web version of PhyML (1,000 transfer bootstrap replicates; http://www.atgc-montpellier.fr/phyml/)^40^. The trees were rooted using the NJ Plot software^41^ and visualized using Interactive Tree of Live (iTOL; https://itol.embl.de/)^42^.

### Identification of HA and NA sequence features

To assess whether the H5 sequences carried markers for mammalian infection, we submitted the nucleotide coding sequences of the H5 isolates to the “Identify Sequence Features in Segments” analysis tool in the Influenza Research Database (https://legacy.fludb.org). Amino acid features with hits are listed for the H5 isolates.

To determine whether our N1 and N2 isolates are susceptible to approved influenza NA inhibitors, we first obtained the amino acid sequences of the N1 and N2 isolates using the EMBOSS Transeq translation tool. The amino acid sequences were then submitted to the “Antiviral Resistance Risk Assessment” analysis tool of the Influenza Research Database.

## Supplementary Information

Supplementary Information.

## Data Availability

All data generated during the current study are included in the manuscript.

## References

[1] Webster RG, Bean WJ, Gorman OT, Chambers TM, Kawaoka Y (1992). Evolution and ecology of influenza A viruses. Microbiol. Rev..

[2] Olsen B (2006). Global patterns of influenza a virus in wild birds. Science.

[3] van Dijk JG, Verhagen JH, Wille M, Waldenström J (2018). Host and virus ecology as determinants of influenza A virus transmission in wild birds. Curr. Opin. Virol..

[4] Swayne DE, Suarez DL (2000). Highly pathogenic avian influenza. Rev. Sci. Tech..

[5] McLeod, A., Morgan, N., Prakash, A., Hinrichs, J. & FAO. *Economic and Social Impacts of Avian Influenza*. https://www.fao.org/3/ag035e/ag035e.pdf (2005).

[6] Hunter P (2022). Europe's worst ever bird flu outbreak: Thus year's epidemic of highly pathogenic avian flu has had a devastating impact on wild and domestic birds and severe economic consequences: Thus year’s epidemic of highly pathogenic avian flu has had a devastating impact on wild and domestic birds and severe economic consequences. EMBO Rep..

[7] Ramey AM (2022). Highly pathogenic avian influenza is an emerging disease threat to wild birds in North America. J. Wildl. Manag..

[8] Centers of Disease Control and Prevention (CDC). *Reported Human Infections with Avian Influenza A Viruses*. https://www.cdc.gov/flu/avianflu/reported-human-infections.htm (2022).

[9] Hoffmann E (2000). Characterization of the influenza A virus gene pool in avian species in southern China: Was H6N1 a derivative or a precursor of H5N1?. J. Virol..

[10] Lee YJ (2008). Highly pathogenic avian influenza virus (H5N1) in domestic poultry and relationship with migratory birds, South Korea. Emerg. Infect. Dis..

[11] Kim HR (2012). Highly pathogenic avian influenza (H5N1) outbreaks in wild birds and poultry, South Korea. Emerg. Infect. Dis..

[12] Jeong J (2014). Highly pathogenic avian influenza virus (H5N8) in domestic poultry and its relationship with migratory birds in South Korea during 2014. Vet. Microbiol..

[13] Lee E-K (2017). Surveillance of avian influenza viruses in South Korea between 2012 and 2014. Virol. J..

[14] Nam JH (2021). Surveillance of avian influenza viruses from 2009 to 2013 in South Korea. Sci. Rep..

[15] Wang W (2010). Glycosylation at 158N of the hemagglutinin protein and receptor binding specificity synergistically affect the antigenicity and immunogenicity of a live attenuated H5N1 A/Vietnam/1203/2004 vaccine virus in ferrets. J. Virol..

[16] Watanabe Y (2011). Acquisition of human-type receptor binding specificity by new H5N1 influenza virus sublineages during their emergence in birds in Egypt. PLoS Pathog..

[17] Kim MK, Lee S-I, Lee SD (2016). Habitat use and its implications for the conservation of the overwintering populations of bean goose *Anser fabalis* and greater white-fronted goose *A. albifrons* in South Korea. Ornithol. Sci..

[18] Cheon S-H (2018). Genetic evidence for the intercontinental movement of avian influenza viruses possessing North American-origin nonstructural gene allele B into South Korea. Infect. Genet. Evol..

[19] Na E-J (2021). Genetic characteristics of avian influenza virus isolated from Wild Birds in South Korea, 2019–2020. Viruses.

[20] Everest H (2020). The evolution, spread and global threat of H6Nx avian influenza viruses. Viruses.

[21] Hu C (2020). Co-circulation of multiple reassortant H6 subtype avian influenza viruses in wild birds in eastern China, 2016–2017. Virol. J..

[22] Yao Z (2022). Genetic and pathogenic characterization of avian influenza virus in migratory birds between 2015 and 2019 in Central China. Microbiol. Spectr..

[23] Ge Y (2017). New H6 influenza virus reassortment strains isolated from *Anser fabalis* in Anhui Province, China. Virol. J..

[24] Ge Y (2017). Epidemic of wild-origin H1NX avian influenza viruses in Anhui, China. Infect. Dis. Poverty.

[25] Wang D (2021). Ecology of avian influenza viruses in migratory birds wintering within the Yangtze River wetlands. Sci. Bull..

[26] Lee YN (2020). Genetic characteristics and pathogenesis of H5 low pathogenic avian influenza viruses from wild birds and domestic ducks in South Korea. Sci. Rep..

[27] Si Y-J (2020). Isolation and characterization of low pathogenic H7N7 avian influenza virus from a red-crowned crane in a zoo in South Korea. BMC Vet. Res..

[28] Lee Y-N (2018). Pathogenesis and genetic characteristics of novel reassortant low-pathogenic avian influenza H7 viruses isolated from migratory birds in the Republic of Korea in the winter of 2016–2017. Emerg. Microbes Infect..

[29] Kim M-K, Lee S-I, Jablonski PG, Lee S-D (2018). Correlation between temperature and the timing of arrival of geese in South Korea. J. Ecol. Environ..

[30] Keeler Shamus P, Dalton Melinda S, Cressler Alan M, Berghaus Roy D, Stallknecht David E (2014). Abiotic factors affecting the persistence of avian influenza virus in surface waters of waterfowl habitats. Appl. Environ. Microbiol..

[31] Duong BT, Bal J, Sung HW, Yeo SJ, Park H (2021). Molecular analysis of the Avian H7 influenza viruses circulating in South Korea during 2018–2019: Evolutionary significance and associated zoonotic threats. Viruses.

[32] Lee YN (2021). Genetic characterization of novel H7Nx low pathogenic avian influenza viruses from wild birds in South Korea during the Winter of 2020–2021. Viruses.

[33] Kwon JH (2018). New reassortant clade 2.3.4.4b avian influenza A(H5N6) virus in Wild Birds, South Korea, 2017–2018. Emerg. Infect. Dis..

[34] World Health Organization (WHO). *Avian Influenza A(H3N8)—China*. https://www.who.int/emergencies/disease-outbreak-news/item/2023-DON456 (2023).

[35] World Organisation for Animal Health (WOAH). *Manual of Diagnostic Tests and Vaccines for Terrestrial Animals 2022*. https://www.woah.org/en/what-we-do/standards/codes-and-manuals/terrestrial-manual-online-access/ (2022).

[36] Hoffmann E, Stech J, Guan Y, Webster RG, Perez DR (2001). Universal primer set for the full-length amplification of all influenza A viruses. Arch. Virol..

[37] Cheung PP (2009). Identifying the species-origin of faecal droppings used for avian influenza virus surveillance in wild-birds. J. Clin. Virol..

[38] Ratnasingham S, Hebert PDN (2007). Bold: The barcode of life data system. Mol. Ecol. Notes.

[39] Higgins DG, Bleasby AJ, Fuchs R (1992). CLUSTAL V: Improved software for multiple sequence alignment. Comput. Appl. Biosci..

[40] Guindon S (2010). New algorithms and methods to estimate maximum-likelihood phylogenies: Assessing the performance of PhyML 3.0. Syst. Biol..

[41] Perriere G, Gouy M (1996). WWW-query: An on-line retrieval system for biological sequence banks. Biochimie.

[42] Letunic I, Bork P (2021). Interactive Tree Of Life (iTOL) v5: An online tool for phylogenetic tree display and annotation. Nucleic Acids Res..

