# Supplementary material for: Surveillance of avian influenza viruses from 2014 to 2018 in South Korea
Source: Sci Rep. 2023 May 24;13:8410. doi: 10.1038/s41598-023-35365-4 (PMC10206579; doi:10.1038/s41598-023-35365-4)
Supplement: Supplementary file 1 — Supplementary Information. [file 41598_2023_35365_MOESM1_ESM.pdf]

SUPPLEMENTARY INFORMATION for

Surveillance of avian influenza viruses from 2014 to 2018 in South Korea

Erica Españo[1], Sang-Mu Shim[1,2], Eun-Jung Song[1,3], Jeong-Hyun Nam[1,4], Seo-Hee Jeong[1], Bill Thaddeus Padasas[1], Sang-Hyun Kim[1], Jeong-Ki Kim[1,*]

[1] *Department of Pharmacy, Korea University College of Pharmacy, Sejong 30019, Republic of Korea*

[2] *Division of Acute Viral Diseases, Center for Emerging Virus Research, National Institute of Infectious Diseases, National Institute of Health, Cheongju, Chungbuk 28159, Republic of Korea*

[3] *Laboratory Animal Medicine, College of Veterinary Medicine, Chonnam National University, Gwangju 61186, Republic of Korea*

[4] *Division of Vaccine Clinical Research, Center for Vaccine Research, National Institute of Infectious Diseases, National Institute of Health, Cheongju, Chungbuk 28159, Republic of Korea*

* Corresponding author: Jeong-Ki Kim; e-mail: jkfrancis@korea.ac.kr; tel.: +82 44 860 1613; fax: +82 44 860 1609

Supplementary Table S1. Distribution of hemagglutinin (HA) subtypes of influenza A viruses isolated from wild bird fecal matter collected in winters of November 2014 to January 2018 in South Korea.

**HA subtypes per year**					
	**2014-2015**	**2015-2016**	**2016-2017**	**2017-2018**	**Total**
**H1**	18	1	1	3	23
**H2**	-	-	-	1	1
**H3**	-	-	1	-	1
**H4**	-	-	-	-	0
**H5**	10	-	3	1	14
**H6**	3	4	11	4	22
**H7**	-	-	2	-	2
**H8**	-	-	5	-	5
**H9**	-	-	-	-	0
**H10**	-	-	-	-	0
**H11**	3	1	-	-	4
**HX**	2	-	1	-	3
**Total**	36	6	24	9	75

Supplementary Table S2. Distribution of different neuraminidase (NA) subtypes of influenza A viruses isolated from wild bird fecal matter collected in winters of November 2014 to January 2018 in South Korea.

NA subtypes					
	**2014-2015**	**2015-2016**	**2016-2017**	**2017-2018**	**Total**
**N1**	15	1	9	7	32
**N2**	5	3	3	1	12
**N3**	14	-	3	-	17
**N4**	-	-	5	-	5
**N5**	-	1	-	1	2
**N6**	-	-	-	-	0
**N7**	-	-	2	-	2
**N8**	-	-	2	-	2
**N9**	1	1	-	-	2
**NX**	1	-	-	-	1
**Total**	36	6	24	9	75

Supplementary Table S3. Distribution of subtypes of influenza A viruses isolated from wild bird fecal matter collected in winters of November 2014 to January 2018 in South Korea.

	**2014-2015**	**2015-2016**	**2016-2017**	**2017-2018**	**Total**
H1N1	12	1	1	3	17
H1N2	2	-	-	-	2
H1N3	3	-	-	-	3
H1N9	1	-	-	-	1
H2N5	-	-	-	1	1
H3N8	-	-	1	-	1
H5N2	1	-	-	1	2
H5N3	8	-	3	-	11
H6N1	1	-	8	4	13
H6N2	2	3	3	-	8
H6N5	-	1	-	-	1
H7N7	-	-	2	-	2
H8N4	-	-	5	-	5
H11N3	3	-	-	-	3
H11N9	-	1	-	-	1
H5NX	1	-	-	-	1
HXN2	2	-	-	-	2
HXN8	-	-	1	-	1

Supplementary Table S4. GenBank accession numbers of the hemagglutinin and neuraminidase coding sequences for the isolates collected in winters of November 2014 to January 2018 in South Korea. Abbreviations: AB, aquatic bird; Gs, goose; Kor, Korea.

	HA	NA
A/AB/Kor/CN57/14 H5N3	OP905456	-
A/AB/Kor/CN58/14 H6N1	OP905457	OP905458
A/AB/Kor/CN59/14 H1N1	OP905459	OP905460
A/AB/Kor/CN60/14 H5N3	OP905461	OP905462
A/AB/Kor/CN61/14 H1N9	OP905463	-
A/AB/Kor/CN62/14 H1N3	OP905464	OP905465
A/AB/Kor/CN63/14 H1N1	OP905466	OP905467
A/AB/Kor/CN64/14 H1N1	OP905468	OP905469
A/AB/Kor/CN65/14 H11N3	OP905470	OP905471
A/AB/Kor/CN66/14 H1N1	OP905472	-
A/AB/Kor/CN67/14 H1N1	OP905473	OP905474
A/AB/Kor/CN68/14 H5Nx	OP905475	-
A/AB/Kor/CN69/14 H6N2	OP905476	OP905477
A/AB/Kor/CN70/14 H1N1	-	OP905478
A/AB/Kor/CN71/14 H5N3	OP905479	OP905480
A/AB/Kor/CN72/14 H5N3	OP905481	OP905482
A/AB/Kor/CN73/14 H5N2	OP905483	OP905484
A/AB/Kor/CN74/14 HxN1	OP905485	-
A/AB/Kor/CN75/14 H1N1	OP905486	OP905487
A/AB/Kor/CN76/14 H1N2	OP905488	OP905489
A/AB/Kor/CN77/14 HxN1	-	OP905490
A/AB/Kor/CN78/14 H1N1	OP935621	OP905491
A/AB/Kor/CN79/14 H5N3	OP905492	OP905493
A/AB/Kor/CN80/14 H5N3	OP905494	OP905495
A/AB/Kor/CN81/14 H1N1	OP905496	OP905497
A/AB/Kor/CN82/14 H1N3	OP905498	OP905499
A/AB/Kor/CN83/14 H1N3	OP905500	OP905501
A/AB/Kor/CN84/14 H5N3	OP905502	OP905503
A/AB/Kor/CN85/14 H1N1	-	OP905504
A/AB/Kor/CN86/14 H1N1	OP935622	OP905505
A/AB/Kor/CN87/14 H6N2	-	OP905506
A/AB/Kor/CN88/14 H11N3	OP905507	OP905508
A/AB/Kor/CN89/14 H1N2	OP905509	OP905510
A/AB/Kor/CN90/14 H11N3	OP905511	OP905512
A/AB/Kor/CN91/14 H1N1	OP905513	OP905514
A/AB/Kor/CN92/14 H5N3	OP905515	OP905516
A/AB/Kor/CN93/15 H6N2	OP905517	OP905518

A/AB/Kor/CN94/15 H6N2	OP905519	-
A/AB/Kor/CN95/15 H1N1	OP905520	OP905521
A/AB/Kor/JB13/16 H6N2	OP905522	OP905523
A/AB/Kor/JB14/16 H6N5	OP905524	OP905525
A/AB/Kor/JB15/16 H11N9	OP905526	-
A/Gs/Kor/CN96/16 H1N1	OP905527	OP905528
A/Gs/Kor/CN97/16 H6N2	OP905529	OP905530
A/Gs/Kor/CN98/16 H6N2	OP905531	OP905532
A/Gs/Kor/CN99/16 H6N1	-	OP905533
A/Gs/Kor/CN100/16 H6N1	OP905534	OP905535
A/Gs/Kor/CN101/16 H6N2	OP905536	OP905537
A/Gs/Kor/CN102/16 H6N1	OP905538	OP905539
A/Gs/Kor/CN103/16 H6N1	OP905540	OP905541
A/Gs/Kor/CN104/16 H6N1	OP905542	OP905543
A/Gs/Kor/CN105/16 H6N1	OP905544	OP905545
A/Gs/Kor/CN106/16 H6N1	OP905546	OP905547
A/Gs/Kor/CN107/16 H3N8	OP905548	-
A/AB/Kor/JB16/16 H6N1	OP905549	OP905550
A/Gs/Kor/JB17/16 H5N3	OP905551	OP905552
A/Gs/Kor/JB18/16 H5N3	OP905553	-
A/Gs/Kor/JB19/16 H5N3	OP905554	OP905555
A/AB/Kor/BS20/17 H7N7	OP905556	-
A/AB/Kor/BS21/17 H7N7	OP905557	-
A/Gs/Kor/BS22/17 H8N4	OP905558	OP905559
A/AB/Kor/BS23/17 H8N4	OP905560	OP905561
A/AB/Kor/CN108/17 H1N1	OP905562	OP905563
A/AB/Kor/CN109/17 H1N1	OP905564	OP905565
A/AB/Kor/JB21/18 H1N1	OP905566	OP905567
A/AB/Kor/JB22/18 H6N1	OP905568	OP905569
A/AB/Kor/JB23/18 H2N5	OP905570	OP905571
A/AB/Kor/JB24/18 H6N1	OP905572	OP905573
A/AB/Kor/JB25/18 H5N2	OP905574	OP905575
A/AB/Kor/JB27/18 H6N1	OP905576	OP905577

Supplementary Table S5. Previously reported influenza A virus isolates with highest similarities with the coding sequences of the isolates from fecal matter collected in South Korea over the winters of November 2014 to January 2018. Abbreviations: AB, aquatic bird; Gs, goose; Kor, Korea.

	HA			NA		
**Sample Name**	**Name**	**Accession***	**%**	**Name**	**Accession***	**%**
A/AB/Kor/CN57/14 H5N3	A/duck/Shimane/321004/2014 (H5N2)	EPI855813	99	-	-	-
A/AB/Kor/CN58/14 H6N1	A/aquatic bird/South Korea/GN25/2013 (H6N2)	OK342167	100	A/aquatic bird/South Korea/SW1/2018 (H10N1)	MK539838	99.29
A/AB/Kor/CN59/14 H1N1	A/duck/Hokkaido/201/2014 (H1N1)	LC339531	99.49	A/duck/Hokkaido/201/2014 (H1N1)	LC3339533	99.79
A/AB/Kor/CN60/14 H5N3	A/duck/Mongolia/107/2014 (H5N7)	LC617425	99.65	A/duck/Tottori/K191/2015 (H5N3)	LC617427	99.86
A/AB/Kor/CN61/14 H1N9	A/Anseriformes/Anhui/L167/2014 (H1N1)	KU881712	99.94	-	-	-
A/AB/Kor/CN62/14 H1N3	A/duck/Kumamoto/431107/2014 (H1N4)	EPI855535	99	A/chicken/Taiwan/01174/2015 (H5N3)	KU646911	99.72
A/AB/Kor/CN63/14 H1N1	A/Anseriformes/Anhui/S3/2014 (H1N1)	KU881672	99.76	A/duck/Hokkaido/201/2014 (H1N1)	LC3339533	99.65
A/AB/Kor/CN64/14 H1N1	A/wild bird/Wuhan/WHHN58/2014 (H1N1)	KU143252	99.77	A/duck/Hokkaido/201/2014 (H1N1)	LC339533	99.58
A/AB/Kor/CN65/14 H11N3	A/crane/Kagoshima/KU-T40/2015 (H11N9)	EPI1057748	98	A/chicken/Taiwan/01174/2015 (H5N3)	KU646911	99.65
A/AB/Kor/CN66/14 H1N1	A/wild bird/Wuhan/WHHN58/2014 (H1N1)	KU143252	99.66	A/duck/Hokkaido/201/2014 (H1N1)	LC3339533	99.82
A/AB/Kor/CN67/14 H1N1	A/wild bird/Wuhan/WHHN58/2014 (H1N1)	KU143252	99.54	A/goose/Inner Mongolia/IM-192/2014 (H5N1)	KR010413	99.57
A/AB/Kor/CN68/14 H5Nx	A/duck/Tottori/Vac-T2/2021 (H5N8)	LC701527	99.65	-	-	-
A/AB/Kor/CN69/14 H6N2	A/duck/Shimane/321004/2014 (H5N2)	EPI855813	99	A/duck/Hokkaido/K04/2014 (H9N2)	LC042045	99.58
A/AB/Kor/CN70/14 H1N1	-	-	-	A/duck/Hokkaido/201/2014 (H1N1)	LC339533	99.58
A/AB/Kor/CN71/14 H5N3	A/wild bird feces/Korea/H2512/2015 (H5N3)	EPI1513754	99	A/goose/Taiwan/01038/2015 (H5N3)	KU646903	99.56
A/AB/Kor/CN72/14 H5N3	A/wild bird feces/Korea/H2512/2015 (H5N3)	EPI1513754	99	A/goose/Taiwan/01038/2015 (H5N3)	KU646903	99.56
A/AB/Kor/CN73/14 H5N2	A/duck/Mongolia/107/2014 (H5N7)	LC011447	99.59	A/duck/Hokkaido/K04/2014 (H9N2)	LC042045	99.86
A/AB/Kor/CN74/14 HxN1	-	-	-	A/Aquatic bird/South Korea/SW1/2018 (H10N1)	MK539838	99.54
A/AB/Kor/CN75/14 H1N1	A/wild bird/Wuhan/WHHN58/2014 (H1N1)	KU143252	99.37	A/goose/Inner Mongolia/IM-192/2014 (H5N1)	KR010413	99.43
A/AB/Kor/CN76/14 H1N2	A/wild bird/Wuhan/WHHN58/2014 (H1N1)	KU143252	99.6	A/duck/Hokkaido/K04/2014 (H9N2)	LC042045.	99.72
A/AB/Kor/CN77/14 HxN1	-	-	-	A/Aquatic bird/South Korea/SW1/2018 (H10N1)	MK539838	99.72
A/AB/Kor/CN78/14 H1N1	A/duck/Hokkaido/201/2014 (H1N1)	LC339531	99.42	A/goose/Inner Mongolia/IM-192/2014 (H5N1)	KR010413	99.43
A/AB/Kor/CN79/14 H5N3	A/duck/Tottori/Vac-T2/2021 (H5N8)	LC701527	99.65	A/wild bird feces/Korea/H2292/2015 (H5N3)	EPI1542785	99
A/AB/Kor/CN80/14 H5N3	A/duck/Tottori/Vac-T2/2021 (H5N8)	LC701527	99.59	A/goose/Taiwan/01038/2015 (H5N3)	KU646903	99.91

A/AB/Kor/CN81/14 H1N1	A/duck/Hokkaido/201/2014 (H1N1)	LC339531	99.59	A/duck/Hokkaido/201/2014 (H1N1)	LC339533	99.43
A/AB/Kor/CN82/14 H1N3	A/wild bird/Wuhan/WHHN58/2014 (H1N1)	KU143252	99.6	A/duck/Pingtung/15010157-1/2015 (H5N3)	MW334144	99.73
A/AB/Kor/CN83/14 H1N3	A/duck/Hokkaido/201/2014 (H1N1)	LC339531	99.54	A/wild bird feces/Korea/H2292/2015 (H5N3)	EPI1542785	99
A/AB/Kor/CN84/14 H5N3	A/duck/Mongolia/107/2014 (H5N7)	LC011447	99.65	A/wild bird feces/Korea/H2292/2015 (H5N3)	EPI1542785	99
A/AB/Kor/CN85/14 H1N1	-	-	-	A/goose/Inner Mongolia/IM-192/2014 (H5N1)	KR010413	99.57
A/AB/Kor/CN86/14 H1N1	A/wild bird/Wuhan/WHHN58/2014 (H1N1)	KU143252	99.6	A/goose/Inner Mongolia/IM-192/2014 (H5N1)	KR010413	99.43
A/AB/Kor/CN87/14 H6N2	-	-	-	A/duck/Hokkaido/K04/2014 (H9N2)	LC042045	99.23
A/AB/Kor/CN88/14 H11N3	A/crane/Kagoshima/KU-T40/2015 (H11N9)	EPI1057748	99	A/duck/Pingtung/15010180/2015 (H5N3)	EPI1891972	99
A/AB/Kor/CN89/14 H1N2	A/wild bird/Wuhan/WHHN58/2014 (H1N1)	KU143252	99.65	A/duck/Hokkaido/K04/2014 (H9N2)	LC042045	99.78
A/AB/Kor/CN90/14 H11N3	A/crane/Kagoshima/KU-T40/2015 (H11N9)	EPI1057748	98	A/duck/Tottori/K191/2015 (H5N3)	EPI1898277	99
A/AB/Kor/CN91/14 H1N1	A/wild bird/Wuhan/WHHN58/2014 (H1N1)	KU143252	99.77	A/duck/Hokkaido/201/2014 (H1N1)	LC339533	99.65
A/AB/Kor/CN92/14 H5N3	A/duck/Shimane/321004/2014 (H5N2)	EPI855813	99	A/duck/Tottori/K191/2015 (H5N3)	EPI1898277	99
A/AB/Kor/CN93/15 H6N2	A/duck/Kyoto/260214/2015 (H6N2)	EPI855615	99	A/duck/Hokkaido/166/2014 (H5N2)	LC371796	98.95
A/AB/Kor/CN94/15 H6N2	A/duck/Kyoto/260214/2015 (H6N2)	EPI855615	99	A/mallard/Korea/M219/2014 (H6N2)	MH130172	99.69
A/AB/Kor/CN95/15 H1N1	A/duck/Mongolia/520/2015 (H1N1)	LC121396	99.65	A/duck/Mongolia/520/2015 (H1N1)	LC121398	99.29
A/AB/Kor/JB13/16 H6N2	A/duck/Kyoto/260214/2015 (H6N2)	EPI855615	99	A/duck/Hokkaido/K04/2014 (H9N2)	LC042045	99.36
A/AB/Kor/JB14/16 H6N5	A/duck/Kyoto/260214/2015 (H6N2)	EPI855615	99	A/duck/Aichi/231002/2016 (H6N5)	EPI866833	98
A/AB/Kor/JB15/16 H11N9	A/Anas falcata/China/D257/2015 (H11N8)	MH547050	99	-	-	-
A/Gs/Kor/CN96/16 H1N1	A/duck/Mongolia/520/2015 (H1N1)	LC121396	99.36	A/duck/Mongolia/520/2015 (H1N1)	LC121398	98.8
A/Gs/Kor/CN97/16 H6N2	A/chicken/Zhejiang/1667/2017 (H6N1)	MG063437.1	99.59	A/white-fronted goose/Korea/F56-3/2017 (H6N2)	MH130148	99.1
A/Gs/Kor/CN98/16 H6N2	A/chicken/Zhejiang/1667/2017 (H6N1)	MG063437.1	99.59	A/white-fronted goose/Korea/F56-3/2017 (H6N2)	MH130148	98.58
A/Gs/Kor/CN99/16 H6N1	-	-	-	A/wild waterfowl/Korea/F14-5/2016 (H6N1)	MH130116	99.28
A/Gs/Kor/CN100/16 H6N1	A/chicken/Zhejiang/1667/2017 (H6N1)	MG063437.1	99	A/wild waterfowl/Korea/F14-5/2016 (H6N1)	MH130116	98.78
A/Gs/Kor/CN101/16 H6N2	A/chicken/Zhejiang/1667/2017(H6N1)	MG063437.1	99.65	A/white-fronted goose/Korea/F56-3/2017 (H6N2)	MH130148	99.19
A/Gs/Kor/CN102/16 H6N1	A/bean goose/Korea/F54-8/2017(H6N1)	MH130130	99.41	A/wild waterfowl/Korea/F14-5/2016 (H6N1)	MH130116	99.41
A/Gs/Kor/CN103/16 H6N1	A/bean goose/Korea/F54-8/2017(H6N1)	MH130130	99.59	A/wild waterfowl/Korea/F14-5/2016 (H6N1)	MH130116	99.57
A/Gs/Kor/CN104/16 H6N1	A/chicken/Zhejiang/1667/2017(H6N1)	MG063437.1	98.94	A/wild waterfowl/Korea/F14-5/2016 (H6N1)	MH130116	99.22
A/Gs/Kor/CN105/16 H6N1	A/wild waterfowl/Korea/F56-1/2017(H6N2)	MH130138.1	98.47	A/duck/Mongolia/520/2015 (H1N1)	LC121398	99.43

A/Gs/Kor/CN106/16 H6N1	A/chicken/Zhejiang/1667/2017(H6N1)	MG063437.1	99.65	A/wild waterfowl/Korea/F14-5/2016 (H6N1)	MH130116	99.01
A/Gs/Kor/CN107/16 H3N8	A/Mallard/South Korea/KNU2019-56/2019 (H3N8)	MW391858	99.3	-	-	-
A/AB/Kor/JB16/16 H6N1	A/chicken/Zhejiang/1667/2017 (H6N1)	MG063437.1	99.41	A/wild waterfowl/Korea/F14-5/2016 (H6N1)	MH130116	99.65
A/Gs/Kor/JB17/16 H5N3	A/mallard/Korea/H125-4/2016 (H5N3)	EPI1513858	99	-	-	-
A/Gs/Kor/JB18/16 H5N3	A/mallard/Korea/H125-4/2016 (H5N3)	EPI1513858	99	A/pintail/Taiwan/WB2478/2017 (H1N3)	MN988823	99.76
A/Gs/Kor/JB19/16 H5N3	A/mallard/Korea/H125-4/2016 (H5N3)	EPI1513858	99	A/mandarin duck/Korea/A44-1-3/2016 (H5N3)	EPI1513881	100
A/AB/Kor/JB20/16 HxN8	-	-	-	A/aquatic bird/South Korea/JN04/2011 (H6N8)	OK342191	100
A/AB/Kor/BS20/17 H7N7	A/red-crowned crane/South Korea/H1026/2017 (H7N7)	MW116746	99.52	A/wild bird/Korea/H922/2017 (H10N7)	EPI1752453	99
A/AB/Kor/BS21/17 H7N7	A/red-crowned crane/South Korea/H1026/2017 (H7N7)	MW116746	99.53	-	-	-
A/Gs/Kor/BS22/17 H8N4	A/duck/Hokkaido/X9/2016 (H8N4)	MK978904	99.41	A/duck/Aichi/231003/2016 (A/H8N4)	EPI866841	99
A/AB/Kor/BS23/17 H8N4	A/duck/Hokkaido/X9/2016 (H8N4)	MK978904	100	A/duck/Aichi/231003/2016 (A/H8N4)	EPI866841	99
A/Gs/Kor/BS24/17 H8N4	A/duck/Hokkaido/X9/2016 (H8N4)	MK978904	99.47	A/duck/Aichi/231003/2016 (A/H8N4)	EPI866841	99
A/AB/Kor/BS25/17 H8N4	A/duck/Hokkaido/X9/2016 (H8N4)	MK978904	99.47	A/duck/Aichi/231003/2016 (A/H8N4)	EPI866841	99
A/AB/Kor/BS26/17 H8N4	A/duck/Hokkaido/X9/2016 (H8N4)	MK978904	99.53	A/duck/Aichi/231003/2016 (A/H8N4)	EPI866841	99
A/AB/Kor/CN108/17 H1N1	A/duck/Mongolia/520/2015 (H1N1)	LC121396	99.14	A/duck/Vietnam/HN4797/2018 (H3N1)	MW936102	99.37
A/AB/Kor/CN109/17 H1N1	A/duck/Mongolia/520/2015 (H1N1)	LC121396	98.85	A/duck/Bangladesh/34191/2017 (H3N1)	MH791653	99.21
A/AB/Kor/JB21/18 H1N1	A/teal/Russia_Primorje/390/2016 (H1N1)	EPI884223	99	A/duck/Bangladesh/34191/2017 (H3N1)	MH791653	99.41
A/AB/Kor/JB22/18 H6N1	A/duck/Hunan/10.27_YYGK57B2-O/2016 (H0)	EPI1834679	99	A/bean goose/Korea/F54-8/2017 (H6N1)	MH130132	99.01
A/AB/Kor/JB23/18 H2N5	A/black-headed gull/Netherlands/4/2016 (H0)	EPI1307122	98	A/Duck/Hokkaido/W24/2020 (H6N5)	MZ414181	99.51
A/AB/Kor/JB24/18 H6N1	A/chicken/Zhejiang/1667/2017 (H6N1)	MG063437	99.12	A/bean goose/Korea/F54-8/2017 (H6N1)	MH130132	99.22
A/AB/Kor/JB25/18 H5N2	A/Duck/Dongting/D76-1/2016 (H5N7)	MF362103	99.29	A/duck/Mongolia/820/2019 (H4N2)	MT020256	99.44
A/AB/Kor/JB27/18 H6N1	A/chicken/Zhejiang/1667/2017 (H6N1)	MG063437	98.89	A/bean goose/Korea/F54-8/2017 (H6N1)	MH130132	99.15

*All accession numbers starting in “EPI” are from GISAID and only have full numerical values for sequence identities.

Supplementary Table S6. Markers for potential mammalian transmission in the HA sequences of H5 isolates. Abbreviations: AB, aquatic bird; Gs, goose; Kor, Korea.

	**Variation***		
**H5 isolate**	**N171**	**A172**	**P251**
A/AB/Kor/CN57/14 H5N3	✓	✓	✓
A/AB/Kor/CN60/14 H5N3	✓	✓	✗
A/AB/Kor/CN68/14 H5NX	✓	✓	✓
A/AB/Kor/CN71/14 H5N3	✓	✓	✓
A/AB/Kor/CN72/14 H5N3	✓	✓	✓
A/AB/Kor/CN73/14 H5N2	✓	✓	✓
A/AB/Kor/CN79/14 H5N3	✓	✓	✓
A/AB/Kor/CN80/14 H5N3	✓	✓	✓
A/AB/Kor/CN84/14 H5N3	✓	✓	✓
A/AB/Kor/CN92/14 H5N3	✓	✓	✓
A/Gs/Kor/JB17/16 H5N3	✓	✓	✓
A/Gs/Kor/JB18/16 H5N3	✓	✓	✓
A/Gs/Kor/JB19/16 H5N3	✓	✓	✓
A/AB/Kor/JB25/17 H5N2	✓	✓	✓

*Numbering is based on the A/Viet Nam/1203/2004 (H5N1) amino acid sequence.

Supplementary Table S7. Host identification using the cytochrome c oxidase subunit 1 (*COI*) gene sequences from wild bird fecal samples collected in the winter of 2016–17 in South Korea. Abbreviations: AB, aquatic bird; Gs, goose; Kor, Korea.

**Isolate**	**Order**	**Family**	**Genus**
A/Gs/Kor/CN96/16 H1N1	Anseriformes	Anatidae	*Anser*
A/Gs/Kor/CN97/16 H6N2	Anseriformes	Anatidae	*Anser*
A/Gs/Kor/CN98/16 H6N2	Anseriformes	Anatidae	*Anser*
A/Gs/Kor/CN99/16 H6N1	Anseriformes	Anatidae	*Anser*
A/Gs/Kor/CN100/16 H6N1	Anseriformes	Anatidae	*Anser*
A/Gs/Kor/CN101/16 H6N2	Anseriformes	Anatidae	*Anser*
A/Gs/Kor/CN102/16 H6N1	Anseriformes	Anatidae	*Anser*
A/Gs/Kor/CN103/16 H6N1	Anseriformes	Anatidae	*Anser*
A/Gs/Kor/CN104/16 H6N1	Anseriformes	Anatidae	*Anser*
A/Gs/Kor/CN105/16 H6N1	Anseriformes	Anatidae	*Anser*
A/Gs/Kor/CN106/16 H6N1	Anseriformes	Anatidae	*Anser*
A/Gs/Kor/CN107/16 H3N8	Anseriformes	Anatidae	*Anser*
A/AB/Kor/JB16/16 H6N1	-	-	-
A/Gs/Kor/JB17/16 H5N3	Anseriformes	Anatidae	*Anser*
A/Gs/Kor/JB18/16 H5N3	Anseriformes	Anatidae	*Anser*
A/Gs/Kor/JB19/16 H5N3	Anseriformes	Anatidae	*Anser*
A/AB/Kor/JB20/16 N8	-	-	-
A/AB/Kor/BS20/17 H7N7	-	-	-
A/AB/Kor/BS21/17 H7N7	-	-	-
A/Gs/Kor/BS22/17 H8N4	Anseriformes	Anatidae	*Anser*
A/AB/Kor/BS23/17 H8N4	-	-	-
A/Gs/Kor/BS24/17 H8N4	Anseriformes	Anatidae	*Anser*
A/AB/Kor/BS25/17 H8N4	-	-	-
A/AB/Kor/BS26/17 H8N4	-	-	-

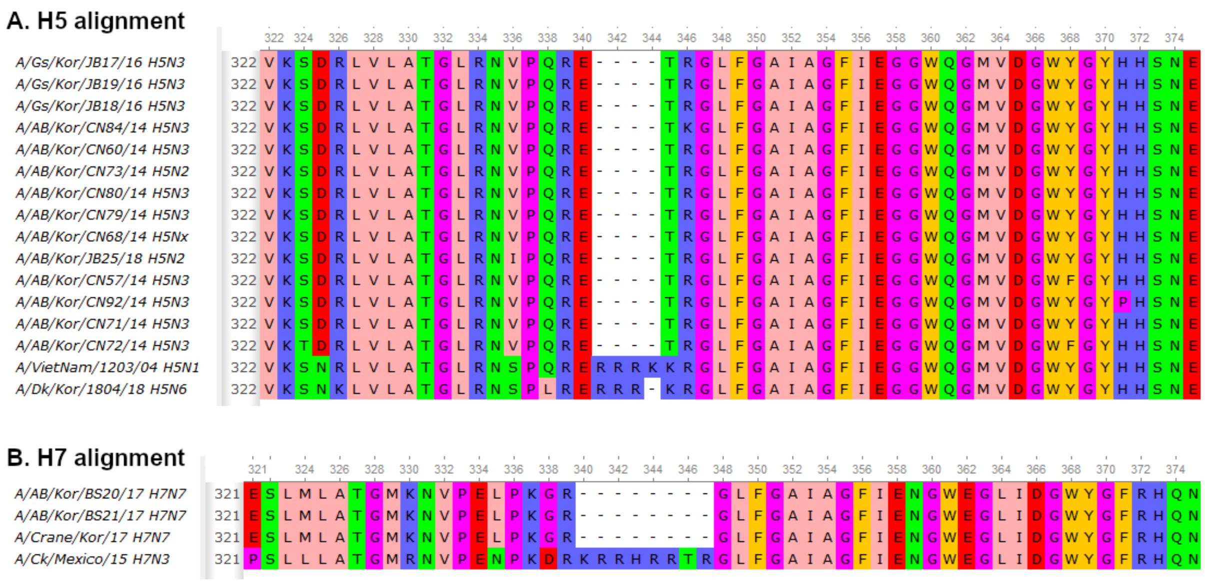

Supplementary Fig. S1. Amino acid sequence alignment sat the hemagglutinin basic cleavage site of the (**A**) H5- and (**B**) H7-subtype isolates in this study with representative highly pathogenic H5 (A/VietNam/1203/04 [H5N1] and A/Dk/Skor/1804 [H5N6]) and H7 (A/Ck/Mexico/15 [H7N3]) influenza viruses. A/Crane/Kor/17 (H7N7) is the closest previously reported match to the H7N7 isolates in our study. Abbreviations: AB, aquatic bird; Ck, chicken; Dk, duck.
